# Application of the OSNA Technique (One-Step Nucleic Acid Amplification Test) in Breast Cancer

**DOI:** 10.3390/ijms26020656

**Published:** 2025-01-14

**Authors:** Grzegorz Sychowski, Hanna Romanowicz, Beata Smolarz

**Affiliations:** Laboratory of Cancer Genetics, Department of Pathology, Polish Mother’s Memorial Hospital Research Institute, Rzgowska 281/289, 93-338 Lodz, Poland; gtspl.3000@gmail.com (G.S.); hanna-romanowicz@wp.pl (H.R.)

**Keywords:** OSNA, breast cancer, metastases, lymph nodes

## Abstract

Breast cancer is one of the most common cancers diagnosed in both countries with high and low levels of socio-academic development. Routine, regular screening tests being introduced in an increasing number of countries make it possible to detect breast cancer at an early stage of development, as a result of which the trend in the incidence of metastatic breast cancer has been decreasing in recent years. The latest guidelines for the treatment of this tumor do not recommend axillary dissection, which limits the need for rapid assessment of the nodes during surgery. Regardless of the progression of the disease, lymph node biopsy and their analysis is one of the most common diagnostic methods for detecting metastases. Systems using one-step amplification of nucleic acids have been present in the diagnosis of breast cancer for nearly 20 years. The one-step nucleic acid amplification (OSNA) test semi-quantitatively detects the number of cytokeratin 19 mRNA copies, a well-known tumor marker, which can be used to infer the presence of metastases in non-sentinel lymph nodes (SLN). **Aim**: OSNA is a widely used molecular method for SLN, intra-, or postoperative analysis. Its high accuracy has been proved over the years in clinical use. In this review, we checked current state of this technology and compared it to its competitors in the field of breast cancer diagnosis in the era of Axillary Lymph Nodes Dissection (ALND) importance decrease with intention to foresee its further potential use. **Objectives**: To evaluate OSNA current place in breast cancer diagnosis and treatment we compared OSNA to other lymph node assessing methods. We based our review on original articles and metanalyses published in the last decade. The research was conducted with PubMed, Science Direct, Google Scholar, and NCBI databases. The collected data allowed us to assess the accuracy of OSNA, its cost effectiveness, and its application in other cancers. **Results**: Regardless of the progression of the disease, a lymph node biopsy and its analysis constitutes one of the most common diagnostic methods for detecting metastases. The OSNA method is characterized by high sensitivity and specificity, and its predictive value has been confirmed by many studies over the years. While its cost effectiveness is still a matter of discussion, this method has been tested more thoroughly than other new lymph nodes assessing technologies. **Conclusions**: Despite the emergence of competing methods, this test is still widely used as a routine intraoperative examination of lymph nodes. Research carried out in recent years has proved its effectiveness in the diagnosis of other cancers, in the research field, and as a provider of additional data for prognosis improvement.

## 1. Breast Cancer

**Figure 1:**
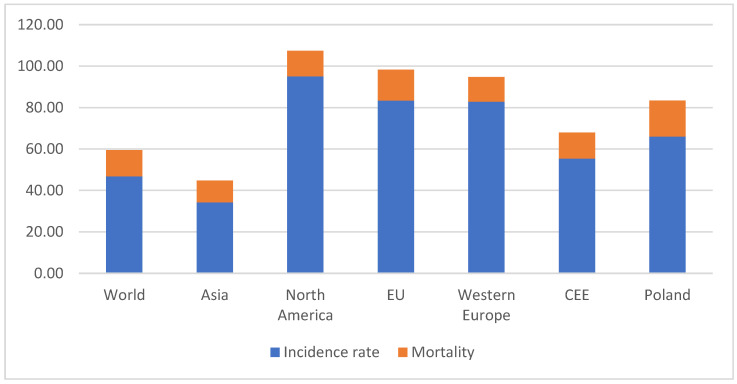
Incidence and mortality rate in age standardized per 100,000 in chosen regions. Data from WHO IARC (World Health Organization, International Agency for Research on Cancer), 2022.

**Figure 2:**
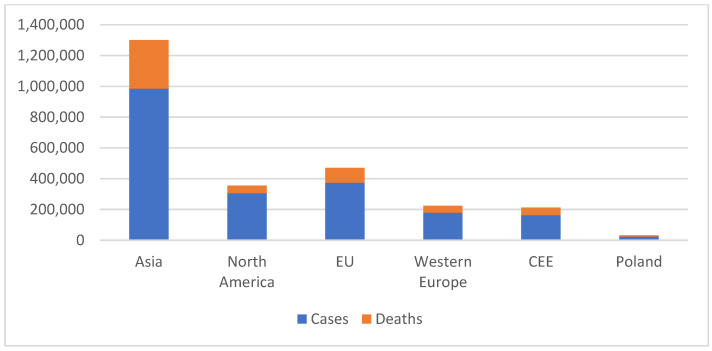
New cases and deaths in 2022 in chosen regions. Data from WHO IARC, 2022.

Breast cancer is one of the most common cancers in women, being nearly ¼ of all diagnosed malignant cancers. In 2022, 2.3 million women worldwide were diagnosed with breast cancer, of whom 670,000 died [1]. According to data from the American Cancer Society, the incidence increases rapidly after the age of 35 in all ethnic groups, although white and black patients in the American population have the highest risk. In Poland, 24% of diagnosed cancers in women in 2021 were breast cancer. Throughout Poland, 21,079 people were diagnosed and 1361 died, and the upward trend is expected to continue in the coming years [2]. Women’s lifetime risk of developing breast cancer is 12.5% (1 in 8); the lifetime risk of dying from breast cancer is 3.6% (1 in 28) [3]. Data on the epidemiology of breast cancer in Western Europe for 2022 include 180,113 new cases (12.7% of all cancers) and 44,387 deaths, which constitute 7.8% of all cancer deaths (four in a row). In Central–Eastern Europe, breast cancer was second only to CRC (Colorectal Cancer) in terms of new cases in 2022. It was diagnosed 163,474 times (12%), of which 49,973 (7.2%) died (3rd in terms of mortality). These numbers are similar to Western Europe, but the mortality rate is higher in absolute terms. This is due to the still lower level of health care in the countries of the former Eastern Bloc, poorer access to screening tests or their quality, and, consequently, initiation of treatment at a higher stage of the disease. Long waiting times for diagnosis and lower availability of drugs also have an impact [4]. In North America, 306,307 (11.5%) new breast cancer (BC) patients were reported in 2022, and 49,744 (7%) died. Despite its more frequent occurrence (95.1 per 100,000 inhabitants compared to 83.4 in the European Union (EU)), the mortality rate is lower than in the European Union (12.3 to 14.9 in the EU). In Asia, BC is the second most common cancer after lung cancer (985,817 cases, 10%) and the 6th cancer in terms of mortality (315,309 deaths, 5.8%) [5]. Above data are presented on Figure 1 and Figure 2, and are summarized in Table 1.

Genetic factors are responsible for 5–10% of cases, the rest is due to higher age, gender, lifestyle, diet, and stimulants used, as these factors affect the hormonal balance [6]. For this reason, taking exogenous estrogen, e.g., in medications or hormonal contraceptives, negatively affects the likelihood of developing cancer. Smoking and the consumption of alcohol and fats yielded similar results, as they contribute to increased estrogen levels, which are associated with a worse prognosis for patients. The research results suggest that for the delay of the first menstrual period and the age of the first birth, the younger the age it occurs, the more it reduces the chance of cancer development. In turn, delaying menopause increases the likelihood of its occurrence—each year is correlated with a 3% greater chance of cancer [7]. The role of the patient’s mental state in the development of the disease is also suggested—depression, long-term stress, the suppression of emotions, and a sense of helplessness are negatively correlated with the health condition and prognosis of patients [8,9]. Breast cancer is characterized by great heterogeneity, and a number of types have been distinguished according to the extent of its development, the morphology and origin of its cells, and the symptoms it presents. For the most part, these are tumors whose cells originate in epithelial tissue, i.e., carcinomas. If the tumor originates from the tissue of the milk ducts or lobules in the breast, it is called ductal or lobular adenocarcinoma. The pre-cancerous state, i.e., the stage of development of the tumor when it occurs only in one place, e.g., in the milk duct, is called ductal carcinoma in situ (DCIS). When it invades the surrounding tissue, it becomes an invasive, infiltrating tumor, which is sometimes called a nonspecific type (NST). Such tumors are most commonly seen in lobules and ducts, and they account for 70–80% of all breast cancers. Along with these conditions, Paget’s disease of the breast is also common (80–90%), causing neoplastic lesions of the nipple and areola. Among invasive carcinomas, triple-negative breast cancer (TNBC), accounting for 15% of all cases, has been singled out for its characteristic cell changes [10]. Its cells are characterized by the absence of estrogen receptors (ERs) or progesterone receptors (PRs) and the lack of production of the HER2 protein (which is coded by *HER2*, an oncogene and a frequent target for therapy), which significantly limits treatment options, as the targets of (targeted) therapies are usually these proteins. It is diagnosed more often in younger premenopausal women, and the disease is often of a higher pT stage. This subtype, together with HER2-negative, has a worse prognosis than other subtypes [11]. Genetic analyses of breast cancer have introduced an additional classification, also called the 3-gene classification, which was created based on the status of ER, PR, and HER2. Luminal A [ER(+) and/or PR(+), HER2(−)]; Luminal B [ER(+) and/or PR(+), HER2(+)], HER2 overexpression [ER(−) and PR(−), HER2(+)], TNBC [ER(−) and PR(−), HER2(−)], and Basal-like—which is a more aggressive type of TNBC often occurring in patients with a BCRA1 mutation. The name comes from the basal cell-like gene expression pattern [12]. The molecular basis of breast cancer is diverse. The tumor usually develops as a result of the impact of the carcinogen directly on the stroma. Whether a microenvironment will be created in which the tumor will be able to develop also depends on macrophages, which have the ability to create an inflammatory environment that promotes angiogenesis and saves cancer cells from immunological rejection. In such an environment, epigenetic factors may also play a role in tumor development, as studies have shown an altered methylation pattern compared to the environment of healthy tissue. The most important genes associated with the development of breast cancer are *BRCA1/2*, *HER2*, *EGFR*, and *c-Myc*, as well as the *Ras* gene family [13,14]. We summarized current data about these genes in Table 2.

Researchers have put forward several theories supported by the research of breast cancer development, which seem to apply to different cases and explain the heterogeneity of this cancer. The stem cell theory suggests that all types of cancer originate from a single line of stem or progenitor cells, which are transformed into the appropriate tumor phenotype only by acquiring mutations. The second theory—stochastic—assumes that the accumulation of mutations in various types of cells leads to their carcinogenesis and the development of the corresponding cancer. Another theory is the evolutionary theory, in which cancer cells arising from mutated stem cells divide to create various tumor cell lines, of which only the most aggressive and adapted to the environment remain and multiply, leading to tumor progression. The development of drug-resistant lines in the tumor may provide a foundation to cause relapses and metastases [15].

## 2. Breast Cancer Diagnostic

Screening allows you to determine the risk or detect the disease before its clinical symptoms appear and is one of the most important preventive methods. There is evidence that such tests may lead to overdiagnosis in the case of breast cancer, which may lead to surgery on tumors that could be treated with less invasive methods. Data prove that such actions do not affect overall survival. The role of the surgeon is to select therapy in such a way as to limit interference in the patient’s body, because each performed surgery is associated with high costs for the health care system and for the patient in financial, physical, and aesthetic dimensions [16]. Performing regular screening tests reduces the risk of death by 20%, which is a strong argument for continuing to carry out screening tests [17]. Breast cancer is typically diagnosed by mammography (MG), ultrasound, or physical examination by a primary care physician [18]. MG is a digital, noninvasive, repeatable, and widely used technique for imaging the structure of the breast using low-dose X-rays. In many countries, it has been introduced in recent decades as a routine screening procedure for patients over 40 years of age. USG, on the other hand, uses ultrasound to obtain a cross-sectional image of tissue. The data indicate higher accuracy of ultrasound compared to mammography, but lower specificity, which is why it is often used together or as a supplement to MG examination, e.g., in cases of high breast density or suspected presence of cysts that are poorly visible in mammograms [19,20]. Other methods supplementing the image from the MG examination are the use of MRI—magnetic resonance imaging—and digital breast tomosynthesis (DBT—3D mammography). Both of these methods generate an accurate 3D image of the breast and are used in high-risk patients. They help in making an accurate diagnosis and reduce the number of visits to the doctor [21]. Breast self-examination is not recommended by specialists and is treated more as an element of prevention than screening. Any changes in breast structure should be a signal for a patient to visit a doctor, who will conduct a clinical breast examination [22].

Breast cancer treatment depends on the stage of the disease, i.e., the size of the tumor, the presence, the number and location of metastases, and the detected markers and their status of ER, PR, or HER2. Based on these factors, the doctor may recommend a breast biopsy (thick or fine needle depending on the type of tumor) in order to obtain a tissue sample from the area around the lesion for further tests. The most frequently used drugs in the treatment of breast cancer are chemotherapy agents, e.g., alkylating agents (Carboplatin), anthracyclines (doxorubicin), or taxanes (Docetaxel). Endocrine therapy involves using aromatase inhibitors (anastrozole) or selective estrogen receptor modulators (Tamoxifen). In immunotherapy, oncologists take advantage of inhibitors (Neratinib) and monoclonal antibodies against ERBB2 (HER2 protein) [18].

To evaluate the tumor or lymph node, a pathological examination is usually performed. Biopsy is the standard procedure in acquiring lymph nodes and is performed by a surgeon. In addition to standard surgical tools such as forceps, dilators and clamps, and a scalpel, a handheld gamma probe equipped with mandatory sentinel targeting is also used for disconnected lymphatic frequencies. Before the procedure, doctors provide the breast area with two staining solutions—one with a radioisotope to locate the tumor with a probe in the body and a blue dye, which has a contrast to the colored node to help the doctor in a detailed location, which is located during its excision by violating while marginally healthy. An alternative to the radioisotope reader is the use of paramagnetic iron oxide power supply (SPIO) (Sienna+) and a magnetic probe (Sentimag) or MRI, fluorescent techniques with ICG (indocyanine green) or fluorescein, or computed tomography of the lymphography or an ultrasound with contrast. These methods differ from each other and the degree of recognition by the medical community. The reduced current tendency to perform lymph node excision at the additional risk of using methods that will not be available in the future or are performed at lower levels on nonrepresentative groups is a consideration [23]. Samples provided by biopsy (aspiration or excision), smear, or in the form of sections are examined by a qualified pathologist for pathological changes in both tissue structure and cells. In the diagnosis of breast cancer, the standard approach is to examine tissue fixed in paraffin (FFPE—Formalin-Fixed Paraffin-Embedded) or frozen in a special medium (FS—Frozen Section), which is faster but less accurate due to the influence of the medium on the image of the tissue. Then, using a microtome, sections of a few µm thick are obtained and placed on microscope slides. Regardless of the fixation method, the preparation is stained to provide contrast to the tissue structures, which helps and enables visual assessment. Most often, these dyes are eosin and hematoxylin, which allow for distinguishing basophilic and acidophilic cell structures. To detect specific proteins or cell components, specific dyes or labeled specific antibodies are used (immunohistochemical staining). Currently, pathological examination is often performed together with the OSNA test or other methods of node assessment.

## 3. OSNA

**Figure 3:**
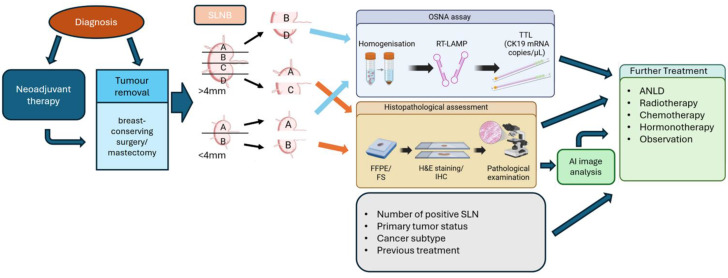
Scheme of procedures in assessment of lymph nodes and the role of OSNA in its process. Created with BioRender [33].

OSNA (one-step nucleic acid amplification) is a method for determining the semi-quantitative amount of cytokeratin 19 mRNA for the intraoperative assessment of the occurrence of metastases in sentinel lymph nodes (SLNs) in breast cancer. A quick assessment of the content of these sequences is possible thanks to the RT-LAMP process—Reverse Transcription Loop-Mediated IsothermalAmplification. This reaction is used in the RD-210 gene amplification detector [23]. It detects the amount of obtained material photometrically, assessing the turbidity of the solution resulting from the amplification process, the by-product of which is the precipitation of magnesium pyrophosphate. Further research on this method has confirmed it as a reliable tool for predicting the chances of further metastases and, consequently, subjecting the patient to more effective therapy. The OSNA system was introduced as a response to the need to shorten the time of intraoperative lymph node examination, which was to determine the need for dissection of further axillary nodes (ALNDs). The aim was to perform SLN and ALND biopsies in one operation to reduce their number, both for health care costs and for the well-being of patients who would not have to be under general anesthesia again [24]. The role of OSNA in process of diagnosis of breast cancer is described in Figure 3. In the field of breast cancer treatment, large, randomized trials have been groundbreaking to evaluate the impact of not performing ALNDs in women with positive axillary SLNs compared with sentinel node dissection (SLND). These were the American College of Surgeons Oncology Group (ACOSOG) Z0011 study—a study conducted on a group of patients with T1-T2 tumors who had one or two positive SLNs and were treated with adjuvant systemic therapy and whole breast irradiation [25], IBCSG 23–01 [26], and ATTRM-048-13-2000 [27]. According to these studies, it is not necessary to perform axillary lymph node dissection if the patient has only local metastases limited to sentinel nodes because it does not affect the overall survival (OS) and disease-free survival (DFS). Studies indicate that metastases occur in only approximately 40–70% of nonsentinel lymph nodes [28]. According to the AMAROS study, the suggested treatment instead of ALNDs is to perform axillary radiotherapy [29]. Moreover, the data even suggest improvement if this procedure is not performed, as it is known to have a negative impact on the patient’s further health and well-being due to frequent complications, e.g., lymphedema and swelling of the arm. Currently, the lower number of ALNDs performed is also due to earlier detection of breast cancer and initiation of treatment before it is necessary [30]. Limiting the number of dissections also reduces the need for intraoperative assessment of the nodes and, consequently, the OSNA system. However, research indicates its great potential for use in other cancers, such as colon, lung, stomach, or endometrium [31]. Despite discussions about its future use, OSNA is still a routine procedure for detecting SLNs during breast cancer surgery in many clinical centers. A meta-analysis of 10,343 nodes from 5,331 patients from 29 studies showed that OSNA is still a highly accurate diagnostic method. Moreover, factors such as ethnicity, the number of diagnosed patients, or the resort in which the tests were performed were not found to have a significant impact on the accuracy of this diagnostic system, which confirms its stability as a diagnostic tool [32].

Cytokeratins belong to the group of the intermediate filament family and have been identified as tumor markers in breast cancer and other cancers of epithelial origin because the cells of this tissue are rich in this type of filament. It has also been found that in the case of neoplastic lesions, cytokeratin mRNA (including CK19) and circulating epithelial cells with metastatic potential can be found in the peripheral blood [34,35]. In the OSNA system, the number of cytokeratin 19 mRNA copies per µL of the tumor sample is determined. The expression level of this gene often changes in cancer cells, which enables the detection of early neoplastic transformation. The literature data indicate that the reduction of CK19 expression in breast cancer is associated with the loss of estrogen and progesterone receptor expression. It is also associated with unfavorable phenotypic features such as a higher tumor stage and a high mitotic index [36]. Increased expression was, in turn, associated with the occurrence of lymph node metastases in thyroid and neuroendocrine tumors. In squamous cell carcinomas, increased CK19 expression was particularly frequently observed on tissue microarrays. Elevated CK19 levels correlated with a higher density of CD8-additional cells in invasive breast cancer, which was most likely related to a stronger immune system response. In turn, the loss of CK19 expression is a common feature of dedifferentiation of cancer cells during its progression [37]. Breast cancer heterogeneity is well pictured in CK19 expression in its subtype cell lines. Luminal subtype cells T47D and MCF7 have high levels of CK19 which expression is correlated with ER receptors. HER2-positive cells SKBR3 and MDA-MB-453 had cytokeratin 19 as well, but in the MDA-MB-231 and MDA-MB-435 cells (triple negative/Claudine low cell line)it was not observed [38,39]. CK19 presence also depends on the stage of the cancer—it was not detected in patients with stage I cancer and benign tumors but in with stage II and III. Moreover, the highest levels were observed in stage II [40]. Alternatively, the research of Shao et al. showed that the expression of CK19 is not significantly linked with tumor grading (over 92% cases had CK19 with different grades). It also confirmed less cases of CK19 expression in triple negative subtype (<80% of cases) and metaplastic carcinoma (<60%) [41]. It was also possible to link the overexpression of CK19 with another important gene in breast cancer—HER2 (human epidermal growth factor receptor 2), which was also overexpressed. HER2 indirectly induces the expression of keratin 19 through the ERk pathway and indirectly through Akt kinase membrane translocation and transition to the granular form. Treatment with anti-CK19 antibodies affects HER2 signaling and disrupts the proliferation of HER2-positive cells [42].

Currently, three main discussions surrounding the OSNA method seem to dominate the literature: (I) The validity of using this research tool and conducting intraoperative node assessment tests; (II) the use of other tools for SLN assessment that will be cheaper, faster, and do not consume research material; and (III) discussion on Total Tumor Load (TTL) cut-off limits that qualify for the diagnosis of micro- and macro-metastases and for ALNDs, with the overall goal of limiting dissections.

## 4. The Validity of Using the OSNA Technique and Performing Intraoperative Nodal Assessment Tests

Sentinel nodes are sometimes collected after neoadjuvant therapy (NAT) conducted to shrink the primary tumor, but such preoperative chemotherapy may confound subsequent diagnosis. Tests using the OSNA method in patients after neoadjuvant therapy were carried out by several independent centers due to doubts as to whether the progression of the disease can be effectively diagnosed when tumor cells are histologically modified with pharmacological agents. However, studies have confirmed the effectiveness of molecular methods such as one-step nucleic acid amplification, as CK19 is still expressed in breast cancer cells after NAT [43]. Compared to traditional histological examination, OSNA detects more micro-metastases, which are easy to miss during visual inspection of the preparations or due to allocation error because they are single modified cells [44]. There was a risk that the very high sensitivity of the OSNA technique could lead to overtreatment of patients and a higher number of dissections, but the study results did not confirm this [45]. There is also no evidence of worse overall disease-free survival (DFS) and overall survival (OS) in patients undergoing OSNA compared to patients undergoing FS (frozen section) and histopathological examination [46,47]. An analysis of breast cancer treatment using OSNA and the ACOSOG Z0011 protocol during the COVID-19 pandemic showed that thanks to the reduced number of repeated LN metastatic surgeries, it was possible to reduce the burden on the health service and start treatment in a larger number of patients [48]. In the case of patients diagnosed with early BC, marking SLN as positive does not currently qualify for extensive axillary treatment, but local targeted biopsy and adjuvant axillary radiotherapy are suggested [49]. According to the latest data, negative lymph node status is not a reason to perform SLNB before neoadjuvant therapy, as it does not affect the overall survival of the patient [50].

## 5. Using Other Tools to Assess SLN That Will Be Cheaper, Faster and Do Not Consume Research Material

Evaluation of the sentinel node for CK19 levels can be assessed by cytokeratin immunohistochemical staining (CK-IHC) intraoperatively from frozen sections or postoperatively from formalin-fixed slides, which appear to have higher accuracy and diagnostic value. The study by Shigematsu et al. showed that the diagnostic abilities of CK-IHC and OSNA are very similar and produce a low rate of false-negative results [46]. This team does not recommend the use of OSNA in patients after NAST (neoadjuvant systemic therapy) because the therapy may disturb gene expression in the examined tissues. This method does not allow pathologists to detect extranodal infiltrates, fibrosis, or scar tissue, which indicate a poor prognosis due to the required homogenization of the lymph node tissue [51]. The direct competition for the OSNA system seemed to be the metasin test, developed within the NHS at Princess Alexandra Hospital in Harlow. Thanks to qRT-PCR, it detected two markers of metastases—CK19 mRNA and mammaglobin relative to the reference gene. Using commonly available reagents and thermal cyclers and lasting approximately 30 min, it seemed to be a promising research tool. According to available data, it is not currently widely used, and its effectiveness is still debated [52,53,54]. The most frequently used method for assessing lymph nodes in the form of section preparations is the analysis of frozen sections, which involves the pathologist assessing the image of the node after its appropriate staining. This technique is relatively cheap and fast, but it is characterized by low sensitivity and high specificity. Unfortunately, the material examined using this technique is often no longer suitable for other procedures and is marked by significant artifacts due to freezing [50]. Intraoperative touch imprint cytology is a so-called touch preparation in which tissue is touched on a slide and leaves its imprint in the form of cells on the microscope slide. The tests are then performed after appropriate staining of the imprinted cells. It is characterized by lower sensitivity than other methods (80–96%) and requires specialized knowledge in the field of cytopathology to evaluate its result [55]; it is also not indicated for diagnosis in early BC [56]. However, it preserves the examined tissue and is associated with lower costs than OSNA [57]. On the contrary, the optical methodsdo not require markers or sample staining at all. One of them is an OCT (optical coherence tomography). It is a technique that uses a polarization-sensitive tomography system and image analysis by algorithms distinguishing healthy from diseased tissue, which can assist doctors in diagnosis [58]. Its current limitations are the lack of sufficientclinical data, cost, and the complexity of measurements [59]. Another technique that uses machine learning to evaluate images is ESS (elastic scattering spectroscopy). It differs from OCT with different imaging equipment and different algorithms, but it also recognizes healthy tissue from pathological lesions in preparations and is currently inferior to other techniques in terms of sensitivity and identification of small metastases [55,59,60]. According to research, artificial intelligence algorithms used in the assessment of H&E-stained slides demonstrate much higher efficiency and sensitivity than pathologists, but their clinical use remains controversial [61]. A promising and very accurate method is infrared spectroscopy with Fourier transform, which allows for the assessment of the biochemical composition of tissue. However, this method is time-consuming and requires prior drying of the preparations. It can potentially be used in plasma-based screening tests [62,63]. A technique that, unlike OSNA, does not consume the test material is the assessment of nodes using Raman spectroscopy. It measures inelastically scattered photons when molecules in a sample are excited by a laser. This technology may also compete with the current gold standard of SLN detection—in vivo tissue staining with double blue dye (BD) and technetium-99 m-labeled nanocolloids. Combined with AT (autofluorescence), it can provide quick results during surgery after administering markers to the patient before the procedure begins. Thanks to this, it additionally creates a map of nodes and outlines of metastatic tumors, which allows for recognizing the size of metastatic foci and making a decision to perform ALND procedures [55]. In the long term, this technique has the potential to replace histopathological examination [64]. At the moment, the method is clinically tested on skin cancers [65], but studies on breast cancer patients using a hand-held fiber-optic probe indicate its great potential [66]. However, the high price of the equipment and relatively long time of data acquisition are still problems to be solved [59]. To objectively compare described methods, their performance was presented in diagnostic odds ratio (DOR) in Table 3. Itis calculated based on NLR and PLR and therefore on sensitivity and specificity, and only these parameters were available for each method, hence the decision to use it as comparative measure. The highest DOR was observed in frozen section method, thanks to its high specifity, and the optical coherence tomography had he lowest values, probably arising from lack of clinical data. The objection to the OSNA method was the fact that the entire node must be examined to avoid allocation error. This results in the irretrievable loss of research material. However, there are studies using this material to analyze gene expression in lymph nodes. The overexpression of genes encoding transcription factors regulating the cell cycle was detected, which allowed for more accurate predictions and personalization of therapy [67].

## 6. Discussion on the Cut-Off Thresholds of TTL That Qualify for the Diagnosis of Micro- and Macro-Metastases and for Performing ALND, with the Overall Goal of Limiting Dissections

Thanks to the OSNA method, we obtained the result of the total number of mRNAs in a given tumor. The result in all examined tumors of a given patient was called TTL (Total Tumor Load), which is an indicator of the degree of SLN involvement [75]. The TTL cut-off limits for the OSNA assay, initially proposed by Tsujimoto et al. in 2007 [76], were 250 copies of CK19 mRNA per μL for determining negative nodes (pN0 patients), 250–5000 copies/μL for micro metastases, and above 5000 copies/μL for macro-metastases, which increased the number of potentially attacked nodes and qualified for ALND examination. Then, in 2013–2014, several independent teams proposed to set the lymph node status prediction upper limit at 15,000 copies/μL [77], 7900 [78], and 7700 [79]. In 2017, the cut-off point distinguishing a positive node from a negative node was set at 2150 copies/μL, which was characterized by a higher degree of specificity, sensitivity, and predictive values than the previous ones. It was also reliable in various breast cancer subtypes [29]. The study by Tomasicchio et al. proposed to use a limit of 9150 copies/μL, but also to base the diagnosis on the TTL value and tumor location, HER2 status, and the number of positive SLNs due to hormonal differences between patients [80]. Since 2019, Sousa’s resarch team successfully used a cut-off point of 30,000 copies/μL, which yielded high false-positive results. A cut-off of 260,000 copies/μL was proposed as a statistically significant predictor of NSLN metastasis [81]. In the case of intraoperative OSNA testing in patients after NAST (neoadjuvant systemic therapy), studies have suggested using TTL values ≥ 15,000 copies/μL to qualify patients for ALNDs, because the use of lower cut-off values does not affect DFS but unnecessarily increases the number of procedures performed. Moreover, it was found that TTL > 25,000 copies/μL indicated a higher risk of disease recurrence [82,83]. Chiloeches et al. also confirmed the validity of using the 15,000-copy limit, but in patients before neoadjuvant therapy. It has been found that any TTL value after NAST qualifies for ALND examination [31].

Independent studies have not recommended the use of OSNA in patients after NAST, but data from studies in recent years suggest otherwise. In 2021, it was found that OSNA is able to diagnose residual disease in patients after NAST [84]. The multi-institutional NEOVATTL study in Italy in 2021 resulted in the creation of an effective predictive model for the risk of NSLN (nonsentinel lymph node) metastasis and disease recurrence in patients undergoing neoadjuvant therapy. This nomogram was based on TTL values in the SLN, primary tumor size, HER2 status (+/−), proliferation index based on the Ki67 protein (>20%), Miller–Payne classification stage, and cN status [85]. The effectiveness of this model has been confirmed by studies on an Italian Spanish cohort, also after NAST and with infiltrating tumors [83], and a study on three populations of Spanish women after NAST and ANLD based on three cut-off points [82]. The effectiveness of the OSNA method after NAST therapy was also confirmed by the research of Pasco Peña et al. due to the lack of observed changes in CK19 expression [86].

An attempt to increase the predictive value of the OSNA method was to introduce the NCS score based on TTL and histological assessment. After removal, the sentinel lymph node was cut into several slices along the short axis and alternately used for OSNA or histological examination. This score has been found to be a strong predictor of nonsentinel metastasis and pN2 status, despite a 10% false-negative OSNA rate due to allocation bias [87].

## 7. Conclusions

One step-nucleic acid amplification analyses number of CK19 mRNA copies/μL in SLN to evaluate and predict the metastases in NSLN. The threshold value determining the need for ANLD has changed over time, and it is still tested for optimal sensitivity and accuracy. Years since its introduction to the market, the OSNA method is still a widely used diagnostic tool, in which potential new applications are still being discovered. Emerging competitive technologies proved to be neither exact enough nor insufficiently tested to be as widely used as one-step amplification method. The high cost per patient issue in OSNA method seem to be still unsolved, despite being brought up numerous times by independent researchers. Homogenized lymph nodes, a byproduct of OSNA test previously treated as waste, turned out to be useful genetic research material. The newest recommendations for lymph node diagnostics and breast cancer treatment suggest a lower demand for intraoperative lymph node evaluation. Regardless of current practices, it seems that in future the OSNA’s exceptional accuracy may find its wider use in postoperative lymph node examination in diagnostics of CK19 positive cancers such as breast, lung, colorectal, endometrium, prostate, cervical or gastric carcinoma and as a research tool. Potential research limitations of this work could be the availability and quality of existing literature, especially the quantity of data from recent years.

## Figures and Tables

**Table 1:** Breast cancer epidemiological data comparison in selected regions. CEE—Central–Eastern Europe. Data from WHO IARC, 2022.

2022	Age Standardized Rates per 100,000		Crude Numbers	
Region	Incidence Rate	Mortality Rate	nr. Cases	Deaths
World	46.0	12.70	2,296,840	666,103
North America	95.10	12.30	306,198	49,727
Asia	34.30	10.50	985,393	315,148
Western Europe	82.80	12.00	180,113	44,387
CEE	55.40	12.50	163,474	49,973
EU	83.40	14.90	375,079	95,881

**Table 2:** Selected most important genes in breast cancer [13,14].

Gene	Coded Protein	Function	Role in Breast Cancer	Expression Status Compared to Normal Cells
*BRCA1*	Breast cancer type 1 susceptibility protein	Oncosuppressor involved in DNA repair processes (double-strand and chromosome breaks) and cell cycle regulation	The accumulation of mutations in this gene results in a predisposition to tumour development—about a 90% chance of developing cancer in carriers of the changed alleles.	Under expression, lowers with disease progression
*BRCA2*	Breast cancer 2; BRCA2, DNA repair associated	Creates protein complex with BRCA1 and RAD51 in Homologous Repair process		Under expression, raised in aggressive tumours
*HER2 (ERBB2)*	Human epidermal growth factor receptor 2	Transmembrane tyrosine kinase, oncogene. Affects cell proliferation and survival.	Strong antiapoptotic and proliferation inducing factor.	Overexpression in 20% of BC cases that require a different treatment process
*EGFR (HER1)*	Epidermal growth factor receptor	Protein-tyrosine kinase. Signalling pathways for DNA synthesis and proliferation.	One of the regulators of cancer proliferation, metastasis, and drug resistance.	Overexpression in aggressive types of BC, i.e., TNBC
*c-Myc*	Transcription factor MYC	Oncogenic transcription factor involved in many pathways including MAKP, mTOR, STAT3	Tumour microenvironment regulator.	Overexpression in advanced, aggressive tumour stages
*Ras* family	H- ras, K- ras, and N- ras	Proto-oncogene with GTPase activity. Involved in cell cycle regulation, growth, migration, apoptosis, and senescence.	Mutation leads to constitutive enzyme activation and induction of hyperproliferation.	Rare mutation in BC
*ESR1/2*	Estrogen receptor	Estrogen activated transcription factor	Allows estrogen to stimulate breast cells to proliferate.	Overexpressed in 70% BC ER+
*PGR*	Progesterone receptor	Progesterone-activated transcription factor	Downregulates ER-α receptor.	High expression in cases with good prognosis (Laminal A)

**Table 3:** Accuracy of described methods. NLR—negative likelihood ratio; values < 1 indicate higher probability of disease if the test was negative. PLR—positive likelihood ratio; the higher the value the more probable is the absence of disease with positive test. DOR—diagnostic odds ratio. Sensitivity and specificity are shown as average from cited sources.

Method	Sensivity	Specifity	NLR	PLR	DOR
OSNA [52,55,68,69]	88.92%	87.43%	0.127	7074	55,819
Metasin test [49,70,71]	92.00%	97.00%	0.082	30,667	371,833
Frozen section histology [55,72]	72.00%	99.70%	0.281	240,000	854,571
Touch imprint cytology [73]	63.00%	98.00%	0.378	31,500	83,432
Raman Spectroscopy (combined with machine learning) [55]	93.00%	95.00%	0.074	18,600	252,429
Fourier transform infrared spectroscopy (FTIR) [62]	95.00%	87.50%	0.057	7600	133,000
Optical Coherent Tomography (OCT) [59,74]	66.70%	79.60%	0.418	3270	7816
Elastic Scattering Spectroscopy (ESS) [55,59]	80.50%	92.50%	0.211	10,733	50,915

## Data Availability

Not applicable.
